# Abstracts from the European Burden of Disease Network: 3rd Working Group Meeting: Virtual. 25-26 January 2022

**DOI:** 10.1186/s13690-023-01034-1

**Published:** 2023-03-31

## A1 Assessing the burden due to non-communicable diseases and injuries: collation of data input sources

### Periklis Charalampous^1^, Elena Pallari^2^, Vanessa Gorasso^3,4^, Elena von der Lippe^5^, Sara M. Pires^6^, Dietrich Plass^7^, Grant M.A. Wyper^8^, Marek Majdan^9^, Brecht Devleesschauwer^4,10^, Juanita A. Haagsma^1^

#### ^1^Department of Public Health, Erasmus MC University Medical Center, Rotterdam, The Netherlands; ^2^Health Innovation Network, Minerva House, Montague Close, London, UK; ^3^Department of Public Health and Primary Care, Ghent University, Ghent, Belgium; ^4^Department of Epidemiology and Public Health, Sciensano, Brussels, Belgium; ^5^Department of Epidemiology and Health Monitoring, Robert Koch Institute, Berlin, Germany; ^6^National Food Institute, Technical University of Denmark, Lyngby, Denmark; ^7^Department for Exposure Assessment and Environmental Health Indicators, German Environment Agency, Berlin, Germany; ^8^Place and Wellbeing Directorate, Public Health Scotland, UK; ^9^Faculty of Health Sciences and Social Work, Trnava University, Slovakia; ^10^Department of Translational Physiology, Infectiology and Public Health, Ghent University, Merelbeke, Belgium

##### **Correspondence:** Periklis Charalampous, p.charalampous@erasmusmc.nl

**Background**

Assessment of the burden of non-communicable diseases (NCDs) and injuries requires high-quality epidemiological data. We aimed to provide an overview of the epidemiological data sources that were used to assess mortality, morbidity, and disability in NCD-specific and injury-specific burden of disease studies undertaken across Europe.

**Methods**

We performed a systematic literature review based on various databases, search engines, and national public health websites, supplemented by hand-searching for burden of disease studies undertaken across European countries. We included burden of disease studies (1990 to 2021) that estimated NCD-specific and injury-specific Years of Life Lost, Years Lived with Disability, and/or Disability-Adjusted Life Years.

**Results**

We extracted data from 89 NCD-specific and 48 injury-specific BoD studies. Over half of the NCD disease burden studies (54%) performed secondary analyses, using the Global Burden of Disease study estimates. Mortality data in independent NCD (53%) and injury (33%) BoD studies were derived from cause-of-death registration systems. Morbidity data on NCDs were obtained from routine administrative and survey databases (52%), whereas morbidity data on injuries were frequently obtained from national injury surveillance systems (61%) – including hospital discharge and emergency department records, or police records. Few independent NCD (33%) and injury (48%) disease burden studies reported the level of completeness of the data systems used.

**Conclusions**

A range of epidemiological data input sources was used to assess the burden of disease due to NCDs and injuries. Reporting of the quality of data input sources can be improved with reporting guidelines and guidelines for evaluating mortality and morbidity data in burden of disease studies.

**Acknowledgements**

This abstract is based upon work from COST Action CA18218 (European Burden of Disease Network; https://www.burden-eu.net/), supported by COST (European Cooperation in Science and Technology; www.cost.eu).

## A2 Disease burden of COVID-19 in the Autonomous Province of Vojvodina, Serbia, from July 2020 to April 2021

### Tijana Lainović^1^, Balázs Ádám^2^, Evgenije Novta^1^, Vesna Mijatović Jovanović^3^, Mioljub Ristić^3^, Vladimir Petrović^3^, Larisa Blažić^1,4^

#### ^1^Faculty of Medicine, School of Dental Medicine, University of Novi Sad, Novi Sad, Serbia; ^2^Public Health Institute, College of Medicine and Health Sciences, United Arab Emirates University, Al Ain, United Arab Emirates; ^3^Faculty of Medicine, University of Novi Sad, Institute of Public Health of Vojvodina, Novi Sad, Serbia; ^4^Dental Clinic of Vojvodina, Novi Sad, Serbia

##### **Correspondence:** Tijana Lainović, tijana.lainovic@mf.uns.ac.rs

**Background**

The COVID-19 hit the world population significantly, causing a great impact on the Burden of Disease (BoD) increase in many countries worldwide. This study aimed to estimate the disease burden of COVID-19 in Vojvodina, the northern province of Serbia, for the period 01/07/2020-14/04/2021.

**Methods**

The overall disease burden caused by COVID-19 in Vojvodina was expressed by Disability Adjusted Life Years (DALYs) metric. The protocol for country studies given by the European BoD Network was used for the burden estimation. The incidence of 65535 new COVID-19 cases was extracted from the Institute of Public Health of Vojvodina database. The gender and age classified cases were divided into 3 groups with different disability weights (DW): 1. COVID–primary health care (DW=0.051); 2. COVID – hospitalized (no intensive care unit (ICU)) (DW=0.133); 3. COVID– ICU support (DW=0.655), as suggested by Burden_EU authors. The unregistered cases, as well as post-covid cases, were not included in the years lived with disability (YLD) estimation, due to insufficient data. Life expectancy for years of life lost (YLL) was taken from the regional life table.

**Results**

The total disease burden was 23 181 DALYs per 1 840 852 population. Expressed per 100 000, the burden was 1259.26 DALYs, with a slightly higher burden in men – 732.05 than in women – 527.21 DALYs. YLD (per 100 000) was 4.54 for females and 4.43 for males, but the YLL was significantly higher in men – 727.62 than in women – 522.67.

**Conclusion**

COVID-19 has been posing an increasing challenge for the health system in Serbia over the past year. DALY metric, composed mainly of the YLL component, depicts well the condition of the affected population.

## A3 PMCardImpact: the health and economic impact of PM2.5-related cardiovascular diseases in Portugal

### Carla Martins^1,2^, Ricardo Assunção^3,4^, Lorena Lima^1^, Mariana Corda^1^, Julian Perelman^1,2^, Florentino Serranheira^1,2^, Dietrich Plass^5^, Ana Timóteo^2,6^, Susana Viegas^1,2^

#### ^1^ NOVA National School of Public Health, NOVA University Lisbon, Lisbon, Portugal; ^2^ NOVA National School of Public Health, Comprehensive Health Research Center, CHRC, NOVA University Lisbon, Lisbon, Portugal; ^3^ IUEM, Instituto Universitário Egas Moniz, Egas Moniz-Cooperativa de Ensino Superior, CRL, Caparica, Portugal; ^4^ Centre for Environmental and Marine Studies (CESAM), University of Aveiro, Aveiro, Portugal; ^5^ Department II 1 Environmental Hygiene, German Environment Agency, Berlin, Germany; ^6^ NOVA Medical School, Universidade NOVA de Lisboa, Lisbon, Portugal

##### **Correspondence:** Carla Martins, carla.martins@ensp.unl.pt

**Background**

Pollution is the largest environmental cause of disease and premature death [1]. The cardiovascular diseases (CVD) and air pollution are linked, and there is evidence of a causal relationship between exposure to particulate matter and cardiovascular morbidity and mortality [2]. Data available in Portugal do not include a comprehensive analysis and the PMCardImpact project aims to overcome this gap with these objectives: to assess the exposure of Portuguese population to PM_2.5_; to estimate the burden of disease and economic impact of PM_2.5_-related CVD in Portugal; and to identify the areas for cost-effective public health interventions.

**Methods**

Data regarding PM_2.5_ levels will be collected from national and European air monitoring platforms. Four scenarios of exposure will be considered for the estimation of burden of disease in Disability-Adjusted Life Years (DALYs) and the economic impact of PM_2.5_-related CVD: i) current scenario; ii) highest PM_2.5_ levels reported; iii) WHO air quality guidelines 2006, and iv) WHO air quality guidelines 2021.

**Results**

A reduction of PM_2.5_ emissions was verified in Portugal over the timeframe 2001–2020. The percentage of hourly exceedance of PM_2.5_ guideline levels has been reduced over the period 2001-2020. However, when considering the recent WHO Air Quality Guidelines, the percentage of exceedances ranged from 40% to 81% over this time. These results under the integrated approach for PM_2.5_ and CVD will be available for application to further pollutants and other environmental risk factors, thus incrementing the potential and the utility of the project.

**Conclusions**

With a view to promote the science to policy interface, PMCardImpact project will make available to policy makers the needed supporting information to act, including actionable knowledge on air pollution trends, related health effects and estimated costs, to implement reducing air pollution policies.

**Acknowledgments**

This work is funded by FCT/MCTES through national funds to PMCardImpact (EXPL/SAU-PUB/0944/2021) and CESAM (UIDP/50017/2020 + UIDB/50017/2020 + LA/P/0094/2020).

**References**

1. Landrigan PJ, Fuller R, Acosta NJR, Adeyi O, Arnold R, Basu N (Nil), et al. The Lancet Commission on pollution and health. Lancet [Internet]. 2018 Feb;391(10119):462–512. Available from: https://linkinghub.elsevier.com/retrieve/pii/S0140673617323450

2. Brauer M, Casadei B, Harrington RA, Kovacs R, Sliwa K. Taking a Stand Against Air Pollution – The Impact on Cardiovascular Disease. Glob Heart. 2021;16(1):1–6.

## A4 Ill-defined deaths by age and sex in Belgrade population, 2016-2020

### Nataša Rosić^1^, Jovana Todorović^2,3^, Aleksandar Stevanović^2,3^ Milena Šantrić-Milićević^2,3^

#### ^1^ City Institute of Public Health Belgrade, Belgrade, Serbia; ^2^Center – School of Public Health, Faculty of Medicine, University of Belgrade, Serbia; ^3^ Institute of Social Medicine, Faculty of Medicine, University of Belgrade, Serbia

##### **Correspondence:** Nataša Rosić, natasa.rosic@zdravlje.org.rs

**Background**

The quality of mortality statistics is closely monitored in national statistical offices and public health institutes. Apart from the system for logic control, an indicator commonly used to signal the quality in coding main causes of death is the share of ill-defined deaths in total mortality [1,2,3]. In this study we focus on the Ill-defined deaths, i.e., deaths coded with the main cause from the XVIII group of ICD-X revision: Symptoms, signs, abnormal findings, ill-defined causes (R00-R99 diagnosis), number of deaths in Belgrade population over the last five years [1,4].

**Methods**

Annual data of ill-defined deaths in Belgrade population are studied by five-year age groups and by sex and the change of the share of ill-defined deaths in total mortality was tracked over the period 2016-2020. Data source the Statistical Office of the Republic of Serbia on the mortality of the citizens of Belgrade [5].

**Results**

**Fig. 1 (abstract A4).**
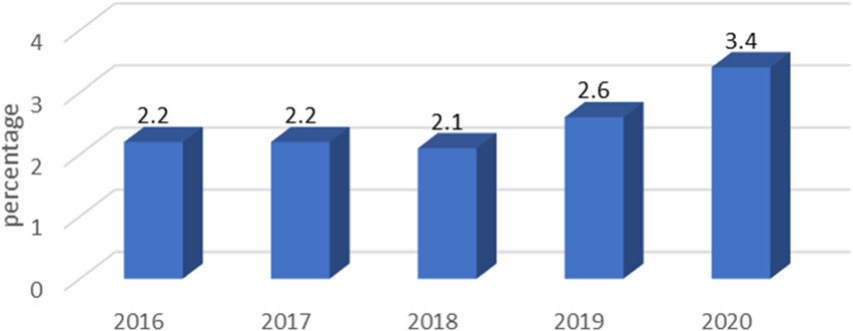
Rank of deceased persons with the diagnosis from the XVIII ICD-X (R00-R99) group in Belgrade between 2016. and 2020

**Fig. 2 (abstract A4).**
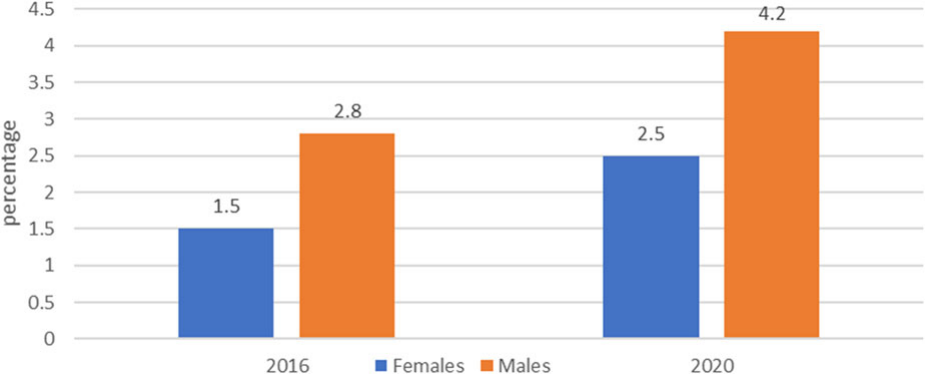
Death due to XVIII ICD-X (R00-R99) group by sex in Belgrade, in 2016 and 2020

The share of ill-defined deaths in total mortality in Belgrade was by one-third higher in 2020 (3.4%), than in period 2016-2019 (2.2%, 2.2%,2.1% and 2.6%, respectively) (Fig. 1). The 2016 v. 2020 increase has equal gender distribution (1.5% v. 2.5% in females and 2.8% v 4.2% in males, respectively) (Fig. 2).

The majority of ill-defined deaths in females and males has moved to older age groups (from 80-84 to 85 years and more and from 60-64 to 65-69 years) most probably due to aging of population.

**Conclusion**

Upgrading the knowledge of physicians responsible for death certification is necessary to decrease the share of ill-defined deaths in the overall mortality.

**References**

1. Nataša Rosić. Unknown and ill-defined causes of death in the mortality of the populations of Serbia, Croatia, North Macedonia and Slovenia, in the period between 2007-2016. Serbian journal of the medical chamber Volume 2 / No. 2 June 2021;23-32.

2. Ylijoki-Sørensen S, Sajantila A, Lalu K, Bøggild H, Lier Boldsen J, Thorup Boel LW. Coding ill-defined and unknown cause of death is 13 times more frequent in Denmarkthan in Finland. Forensic Science International 2014;(244): 289-94.

3. Kulhánová I, Menvielle G, BoppM, Borrell C, Deboosere P, Eikemo TA, et al. Socioeconomic differences in the use of ill-defined causes of death in 16 European countries. BMC Public Health 2014;14:1295.

4. Tin Oung M, Richter K, Prasartkul P, Tangcharoensathien V. Myanmar mortality registration: an assessment for system improvement. Popul Health Metr. 2017 Sep 25;15(1):34.

5. Republika Srbija. Republički zavod za statistiku, Vitalni događaji 2020.

## A5 Differences between the registered and expected mortality in Serbia in 2020

### Aleksandar Stevanović^1,2^, Jovana Todorović^1,2^, Nataša Rosić^3^, Gordana Bjelobrk^4^, Milena Šantrić-Milićević^,1,2^

#### ^1^Institute of Social Medicine, Faculty of Medicine, University of Belgrade, Belgrade, Serbia; ^2^Center – School of Public Health, Faculty of Medicine, University of Belgrade, Belgrade, Serbia; ^3^City Institute of Public Health Belgrade, Belgrade, Serbia; ^4^Statistical Office of the Republic of Serbia, Belgrade, Serbia

##### **Correspondence:** Aleksandar Stevanović, aleksandar.stevanovic@med.bg.ac.rs

**Background**

The impact of the COVID-19 pandemic on the global and local mortality structure has attracted the experts’ attention [1,2]. The officially registered number of deaths in the countries’ vital statistics is used to estimate the COVID-19 mortality burden.

**Methods**

Mortality data disaggregated by cause and sex were acquired from the Statistical Office of the Republic of Serbia. The expected number of deaths (M_E_) in 2020 was derived as a linear trend for the previous 5-year period (2015 to 2019), and then compared to the registered number of deaths (M_R_) for the same year. Absolute and relative changes in contribution of different causes of death to the all-cause mortality were calculated based on the difference between the registered and the expected number of deaths (M_R_ – M_E_).

**Results**

Registered all-cause mortality in 2020 was 116,850, which was 13.5% higher than expected (102,993). Despite the absolute increase in the number of deaths, relative contribution to the all-cause mortality decreased or stagnated in fourteen diagnostic groups. Excluding COVID-19 codes, the absolute difference between the registered and the expected number of deaths (M_R_ – M_E_) was the largest for the disease group of the circulatory (1571, +2.9%) and respiratory system (1455, +27.7%).

**Conclusions**

COVID-19 is the third leading cause of death with 6629 and 3727 fatalities in males and females, respectively. The relative contribution of the leading causes of death (circulatory system diseases and neoplasms) decreased in both sexes. This study provides an orientation for further research needed to determine the impact of COVID-19 on the society.

**References**

1. Islam N, Shkolnikov VM, Acosta RJ, Klimkin I, Kawachi I, Irizarry RA, et al. Excess deaths associated with covid-19 pandemic in 2020: Age and sex disaggregated time series analysis in 29 high income countries. BMJ. 2021;

2. Weinberger DM, Chen J, Cohen T, Crawford FW, Mostashari F, Olson D, et al. Estimation of excess deaths associated with the COVID-19 pandemic in the United States, March to May 2020. JAMA Internal Medicine. 2020;180(10):1336.

## A6 Burden of breast, cervical and colorectal cancers in the countries of former Yugoslavia

### Jovana Todorović, Milena Šantrić-Milićević, Željka Stamenković, Aleksandar Stevanovic, Zorica Terzic Supic

#### Institute of Social Medicine, Faculty of Medicine, University of Belgrade, Belgrade, Serbia

##### **Correspondence:** Jovana Todorović, jovana.todorovic@med.bg.ac.rs

**Background**

Breast cancer, cervical cancer and colorectal cancer are all part of established screening programs and can be diagnosed and treated in the early stages of the disease [1-4]. The aim of this study was to examine the trends in burden of breast cancer, cervical cancer and colorectal cancer in countries of the former Yugoslavia in the period between 1990 and 2019.

**Methods**

The current study focused on the trends of disability-adjusted life years (DALY) using the global burden of disease (GBD) database, and to forecast the burden of these cancers in 2030 in these countries. The analysis was conducted using the estimates for 2019 GBD study available from the Institute of Health Metrics and evaluation. The data used were on the DALYs. The data were presented as rates per 100,000.

**Results**

The highest burden of breast cancer in 2019 was in Serbia (670.84/100000), as was the highest burden of cervical cancer (283.47/100000), while the highest burden of the colon and rectum cancer was in Croatia (1044.32/100000). The highest forecasted burden for 2030 for the breast and cervical cancers is for Serbia, while the highest forecasted burden for colon and rectum cancers is for Croatia, although the burden of this cancer is expected to rise in all former Yugoslavian countries except in Slovenia.

**Conclusions**

The burden of breast, cervical and colon and rectum cancers is significant in the countries of former Yugoslavia.

**References**

1. Beaber EF, Kim JJ, Schapira MM, Tosteson ANA, Zauber AG, Geiger AM, et al. Unifying screening processes within the PROSPR consortium: A conceptual model for breast, cervical, and colorectal cancer screening. J Natl Cancer Inst. 2015;107(6):1–8.

2. Abbafati C, Abbas KM, Abbasi-Kangevari M, Abd-Allah F, Abdelalim A, Abdollahi M, et al. Global burden of 369 diseases and injuries in 204 countries and territories, 1990–2019: a systematic analysis for the Global Burden of Disease Study 2019. Lancet. 2020;396(10258):1204–22

3. Collaboration GB of DC, Fitzmaurice C, Abate D, Abbasi N, Abbastabar H, Abd-Allah F, et al. Global, Regional, and National Cancer Incidence, Mortality, Years of Life Lost, Years Lived With Disability, and Disability-Adjusted Life-Years for 29 Cancer Groups, 1990 to 2017: A Systematic Analysis for the Global Burden of Disease Study. JAMA Oncol. 2019 Sep;5(12):1749–68.

4. Global, regional, and national disability-adjusted life-years (DALYs) for 359 diseases and injuries and healthy life expectancy (HALE) for 195 countries and territories, 1990-2017: a systematic analysis for the Global Burden of Disease Study 2017. Lancet (London, England). 2018 Nov;392(10159):1859–922.

## A7 CATINCA - Capacities and infrastructures for health policy development in South Caucasus and Central Asia. Strengthening information systems for summary measures of population health

### Aline Anton^1^, Alexander Rommel^1^, Katarzyna Kissimova-Skarbek^1, 2^, Caoimhe Cawley^1^, Elena von der Lippe^1^

#### ^1^Affilliation 1: Department 2: Epidemiology and Health Monitoring, Robert Koch Institute, Berlin, Germany; ^2^Affilliation 2: Institute of Public health, Jagiellonian University Medical College, Krakow, Poland

##### **Correspondence:** Aline Anton, antona@rki.de

**Background**

Most countries of Central Asia/Southern Caucasus though part of WHO Europe are ineligible for funding in EU networks like burden-EU COST. Thus, the overarching aim of CATINCA is to involve this region in burden-EU COST by establishing an affiliated network. Specific objectives are capacity building, to enhance the use of existing data, and to contribute to the development of health information systems.

**Methods**

Led by the Robert Koch Institute, CATINCA is funded by the German Ministry of Education and Research (2021-2024). In a trilateral exchange between Germany, burden-EU COST and the partners, it concentrates on calculations of burden of disease (BoD) and other summary measures of population health. CATINCA is intended to achieve its objectives through (i) needs assessment (ii) training and workshops (iii) support of doctoral/master theses (iv) scientific missions (vi) participation in burden-EU COST activities and (vii) pilot studies.

**Results**

8 partners from Mongolia, Kyrgyzstan, Georgia, Kazakhstan, Azerbaijan, Uzbekistan, Armenia and Tajikistan have agreed to actively participate in CATINCA. In a needs assessment that was done in four kick-off meetings, forms of cooperation were agreed, potential topics identified and an overview of exisiting data sources was compiled. As BoD methodology is mostly new in the target countries, the partners consistently expressed a high need for training. Some countries are aiming for piloting analyses on the burden of diabetes, cancers and COVID-19. A first training on basic concepts is planned for spring 2022.

**Conclusion**

Summary measures of population health are equally important for international comparisons and priority setting at the national level. CATINCA helps to implement BoD methodology as an important tool for health information and policy development.

## A8 BoCO-19: The Burden of Disease due to COVID-19. Towards a harmonization of population health metrics for the surveillance of dynamic outbreaks

### Caoimhe Cawley^1^, Elena von der Lippe^1^, Aline Anton^1^, Milena Santric Milicevic^2^, Natalya Glushkova^3^, Kairat Davletov^3^, Alexander Rommel^1^

#### ^1^Department of Health Monitoring and Epidemiology, Robert Koch-Institute, Berlin, Germany; ^2^Faculty of Medicine, University of Belgrade, Belgrade, Serbia; ^3^The Faculty of Medicine and Healthcare, Kazakh National Medical University, Almaty, Kazakhstan

##### **Correspondence:** Caoimhe Cawley, CawleyC@rki.de

**Background**

The BoCO-19 Project (led by the Robert Koch Institute, with support from the burden-EU COST Action) aims to develop and implement harmonized methods for calculation of the burden of disease (BoD) due to COVID-19, working with 14 partner institutions in countries in South-East Europe, the Caucasus and Central Asia.

**Methods**

BoCO-19 consists of four work packages (WP). The first WP established the scientific network and mechanisms for collaboration. The second WP will collect and prepare input data in a harmonized format, and develop a common methodology. A cross-country analytical approach has been developed combining basic analyses (duration/severity assumed globally) with estimates over time and at the sub-national level. More in-depth country-specific analyses are also possible (e.g. country specific severity). BoD calculations will be conducted by the partners, aggregated for comparative analyses (WP3) and disseminated (WP4).

**Results**

Collaboration mechanisms have been established and an initial assessment of data availability in each country has been made. A baseline workshop was held in November 2021 - this included training on BoD methods as well as discussion of methodological challenges and possible solutions. Three Working Groups have been created; these will focus on i) preparation of a standardized data collection template, ii) more detailed discussions of methodological challenges and solutions, and iii) publications/dissemination of information. Two data labs are planned for 2022, during which the burden of COVID-19 in each country will be calculated and compared.

**Conclusions**

Harmonized methodologies are required for timely calculation and international comparison of the burden of COVID-19. BoCO-19 may act as a blueprint for the integration of BoD-indicators into the surveillance of future pandemics and dynamic outbreaks.

## A9 Social and economic burden of chronic diseases in Bulgaria - a case with multiple sclerosis and asthma

### Zornitsa Mitkova, Yoana Seitaridou, Maria Kamusheva, Maria Dimitrova, Petya Milushewa, Guenka Petrova

#### Department of Organization and Economy of Pharmacy, Faculty of Pharmacy, Medical University Sofia, Sofia, Bulgaria

##### **Correspondence:** Zornitsa Mitkova, zmitkova@pharmfac.mu-sofia.bg

**Background**

Implementation of National Prophylactic Programs could prevent many chronic diseases. The aging population in Europe is a main reason for high burden of chronic disease and healthcare costs growth. [1,2] The chronic diseases have also great social burden, especially in low- and middle-income countries mainly due to the limited number of prevention measures.

**Methods**

A retrospective analysis was performed on the basis of real-world data for two groups of patients with high prevalent and socially significant chronic diseases in Bulgaria: asthma and multiple sclerosis (MS). On total 68 patients with asthma and 230 with MS were included in the analysis. Disability weights for asthma and MS were assumed from WHO methodology. Additionally, the average indirect costs were calculated using the mean Gross Domestic Product (GDP) per capita value for the period 2009-2019 which was equal to 6,402.72 euro.

**Results**

The individual *disability-adjusted life years (DALYs)* varied significantly among the patients. The average value of *years of healthy life lost due to disability (YLD*) for asthma was 0.6562 and for MS – 3.63. For both groups *years of life lost (YLLs)* were equal to zero because there was no information about deaths among the patients for the observed period. The average DALYs and indirect costs were 0.6562 and 4,030.00 euro per patient with asthma, and 3.63 and 23267 euro per MS patients.

The comparison of calculated indirect costs (Table 1) reveals that they are much higher in patients suffering from MS.

**Conclusion**

The current study revealed high indirect costs thus illustrating the high economic burden of asthma and MS in Bulgaria. Overall health of chronic patients could be affected by the low incomes, the lack of prevention programs and delayed time to diagnostics.

**References**

1. Mitkova Z, Doneva M, Gerasimov N, Tachkov K, Dimitrova M, Kamusheva M, Petrova G. Analysis of Healthcare Expenditures in Bulgaria. *Healthcare.* 2022; 10(2):274. https://doi.org/10.3390/healthcare10020274

2. Ali A, Sayed M. Determinants of Healthcare Expenditures in GCC Countries: A Panel Data Analysis. J. Asian Financ. Econ. Bus. 2020; 7: 705–714

**Table 1 (abstract A9).** Comparison of indirect costs from different point of view

	Asthma patients	MS patients
Average indirect cost, euro	4030	23267.03
Min - max indirect cost, euro	68.8293 - 11012.69	5263.04 -76314.1
Average period of diagnostics, years	14.93	8.84
Female / male average indirect cost, euro	4267/ 4340	23621/22441

## A10 Case study: Understanding the economic impact of COVID-19 on health systems

### Neil Chalmers^1^, João Vasco Santos^2,3^, Diana Alecsandra Grad^4^, Grant Wyper^1^

#### ^1^Public Health Scotland: Place and Wellbeing, Edinburgh, Scotland, UK; ^2^MEDCIDS – Department of Community Medicine, Information and Health Decision Sciences, Faculty of Medicine, University of Porto, Portugal; ^3^Public Health Unit, Aces Grande Porto V - Porto Ocidental, ARS Norte, Portugal; ^4^ Department of Public Health, Faculty of Political, Administrative and Communication Sciences, Babes-Bolyai University, Cluj-Napoca, Romania

##### **Correspondence:** Neil Chalmers, neil.chalmers@phs.scot

**Background**

Estimating the economic impact of COVID-19 in terms of Global Burden of Disease (GBD) DALYs on health systems in various European countries with respect to health expenditure is important for understanding some of the economic impact of the COVID-19 pandemic and will help for future pandemics, system resilience and financial planning.

**Case report**

This case report/study will essentially collate different GBD DALYs and health expenditure data from across Europe which should then provide inference on the research goal: “*Estimating the economic impact of COVID-19 on health systems in various countries in terms of health expenditure*”.

Existing reports from Audit Scotland (2021) state that *“£1.67 billion in costs associated with Covid-19 for 2020/21*” [1]. These forms of existing studies will help form this ongoing case study.

**Conclusion**

This international collaboration will provide capacity building for future pandemics and for increasing capacity of applying health economics within a burden of disease framework.

Understanding health expenditure data and its relation to DALYs in terms of the COVID-19 pandemic is an important area, as it incorporates costing information on both disability and mortality, which has received little attention within the current literature.

Comparing the health expenditure and the health outcomes in terms of DALYs across the different countries will likely provide useful inference for future pandemics and potential health outcomes based on health expenditure data.

**Reference**

1. Audit Scotland (2021). “Report: NHS in Scotland 2020”. https://www.audit-scotland.gov.uk/report/nhs-in-scotland-2020 (Accessed 13/01/21)

